# Analyzing the Effect of Microbial Consortia Fermentation on the Quality of HnB by Untargeted Metabolomics

**DOI:** 10.4014/jmb.2402.02039

**Published:** 2024-07-30

**Authors:** Ling Zou, Hong Zhang, Zhonghua Liu, Jianfeng Sun, Yang Hu, Yishu Ding, Xinwei Ji, Zhenfei Yang, Qi Zhang, Binbin Hu

**Affiliations:** 1 Yunnan Academy of Tobacco Agricultural Science, Kunming 650021, Yunnan, P.R. China; 2 Faculty of Life Science and Technology, Kunming University of Science and Technology, Kunming 650500, Yunnan, P.R. China; 3 China National Tobacco Corporation Yunnan Company, Kunming 650032, P.R. China; 4 Chuxiong Prefecture Branch of Yunnan Tobacco Company, Chuxiong 675000, P.R. China; 5 Honghe Prefecture Branch of Yunnan Tobacco Company, Honghe, P.R. China

**Keywords:** Microbial consortia, fermentation, HnB cigarettes, metabolomics, mechanisms

## Abstract

Fermentation has been identified as an effective strategy to alter the chemical makeup of tobacco, thereby enhancing its quality. The deliberate introduction of microorganisms can hasten the fermentation process. In this research, microbial consortia harvested from the tobacco surface were utilized to enhance the tobacco quality. This enhancement also elevated several sensory attributes of HnB cigarettes, such as aroma richness, moisture, strength, and reduced irritation, achieving a sensory quality rating of 84.5. This marks a notable improvement compared to the 82 rating of the original, unfermented cigarettes. Untargeted metabolomics analysis revealed a decrease in total polyphenols and unsaturated fatty acids, while the levels of polyacids, alcohols, ketones, furans, and other compounds increased in the fermented tobacco. Additionally, KEGG pathway enrichment analysis indicated that the enhancement in tobacco quality through microbial consortia fermentation is linked to various biological pathways, with pathways related to fatty acid and amino acid degradation playing pivotal roles. The findings of this study will serve as a reference for the commercial production of HnB cigarettes, and the elucidated mechanism offers a theoretical basis for exploring microbial fermentation as a means to improve tobacco quality.

## Introduction

Heat-not-burn (HnB) cigarette products are a new type of tobacco product that utilizes a heating device to treat tobacco material at lower temperatures to produce aerosols for consumers to smoke [1, 2], but their taste is close to that of traditional cigarettes. The low temperature heats at approximately 350°C rather than burning at approximately 600°C [3, 4], which means that the harmful substances in HnB cigarettes will decrease by more than 90% compared to traditional burning cigarettes [5]; therefore, HnB cigarettes have gradually become popular with consumers and now occupy an important position in the international market. HnB cigarettes are made from modulated raw tobacco and some flavor additives in a certain proportion, of which raw tobacco is still the main component. However, HnB cigarettes fail to release more aromatic substances from the tobacco itself through burning, as traditional cigarettes do, since the raw tobacco used at present is poorly adapted to HnB cigarettes [6]. Therefore, effective fermentation measures must be taken to improve the quality of the raw material used for tobacco to enhance the aroma of HnB cigarettes.

Flue-cured tobacco, as the main raw material for HnB cigarettes, has a complex chemical composition that includes alcohols, aldehydes, ketones, phenols, esters, acids, etc., and many types of tobacco aroma substances [7, 8]; additionally, the quality of HnB cigarettes is often directly determined [9]. Aroma substances are affected by fermentation [10], and only fermented flue-cured tobacco, which is less contaminated and has a stronger flavor, can be used for industrial production. Natural fermentation is still generally used in factories but usually takes 1–3 years and does not work well, which is not conducive to industrial development [11]. However, artificial fermentation effectively shortens the fermentation period, improves the quality of tobacco, and improves the quality and stylistic characteristics of tobacco for different cigarette products [12]. Artificial fermentation increases the activity of tobacco surface enzymes and optimizes the microbial community structure, resulting in a significant increase in fermentation efficiency [13]. With the development of microbiomics technology, researchers have shown that the microbial community on the surface of tobacco leaves changes significantly after fermentation, which means that the microorganisms attached to the surface of tobacco leaves truly promote the process of tobacco fermentation [14, 15].

The essence of microbial fermentation in tobacco lies in the application of microorganisms and microbial enzymes to interact with the chemical components within the tobacco leaf. This interaction leads to the transformation of macromolecules such as starch, cellulose, and pectin into precursors for aroma and volatile aroma substances [16, 17]. This process facilitates the discovery of functional microorganisms, the analysis of the evolutionary mechanism of microbial communities, and the extensive promotion of the industrial application of microbial fermented tobacco [18]. The fermenting microorganisms utilized in the present study are derived from the tobacco itself, primarily bacteria. This includes microorganisms from the tobacco surface, tobacco soil, and those from reconstituted tobacco concentrates [19]. However, microorganisms from tobacco fields and reclaimed tobacco are found in more complex environments, affecting their activity and thus, the consistency of fermentation quality cannot be guaranteed [20]. Therefore, the use of microorganisms isolated from the surface of tobacco leaves for fermentation presents the most straightforward and effective method, emerging as a prominent area of research.

There have been several reports on the isolation of strains from the surface of tobacco leaves and their application in tobacco fermentation. The quality grade of tobacco has seen significant enhancement post-fermentation by *Bacillus subtilis* [21]. *Bacillus subtilis* has been shown to reduce the content of macromolecules such as cellulose and starch in flue-cured tobacco [22]. Other microorganisms such as *Pseudomonas*, *Pantoea*, *Burkholderia*, *Monographella*, *Aspergillus*, and *Vibrio* have also been effective in improving tobacco quality [14, 23, 24]. Furthermore, *Bacillus amyloliticus* and *Bacillus kochii* have demonstrated increased effectiveness in fermentation when applied together on tobacco leaves [12]. Based on these findings, it is believed that adding microbial consortia from the surface of the tobacco leaf is more beneficial for enhancing tobacco quality, and a combination of microorganisms may yield a more significant effect. While research on microbial fermentation of tobacco has predominantly focused on cigarillos [25], natural fermentation has been mainly applied in studies of flue-cured tobacco. Thus, this study employed microbial consortia obtained through enrichment and domestication from the surface of the tobacco leaf which contain *Cronobacter* (64.87%), *Bacillus* (10.1%), *Franconibacter* (17.01%) and others unidentified cyanobacteria to ferment flue-cured tobacco. Metabolomics was used to analyze the mechanism by which microbial consortia affect the quality of the tobacco leaf. Additionally, the flue-cured tobacco materials were processed into HnB cigarettes without other ingredients for sensory evaluation. The results will offer theoretical guidance for the industrial production of HnB cigarettes and a theoretical reference for the study of microbial enhancement of tobacco quality mechanisms.

## Materials and Methods

### Tobacco Samples and Strains

In this study, KRK26, a flue-cured tobacco variety grown in Yanshan County, Yunnan Province, was used as fermentation material. After being subjected to flue-curing, middle tobacco leaves were collected, pulverized, and sieved through a 200-mesh sieve at low temperature, after which the powder obtained was subjected to fermentation. The strains used for fermentation were microbiota consortia obtained by enrichment and domestication from the surface of tobacco leaves, which contain *Cronobacter* (64.87%), *Franconibacter* (17.01%), *Bacillus* (10.1%), *cyanobacteria* (2.83%) and others microorganisms.

### Fermentation of Tobacco

Each strains strored in glycerol tubes was inoculated into LB solid media for activation, inoculated into LB liquid media, and cultured at 37°C for 20 h until the OD_600_ reached 1.8–2.0. The colonies were collected at this point, washed, and resuspended in sterile deionized water to obtain the bacterial suspension. The bacterial suspension was added to the tobacco powder at a ratio of 0.4 ml/g, mixed well, sealed, and incubated at 37°C for 7 days of fermentation. The samples at 0, 3, and 7 days of fermentation were collected for sensory evaluation and untargeted metabolomics analysis. Three replicates of samples were set up for each group. The different samples are denoted by EM-0, EM-3 and EM-7.

### Assessment of Sensory Quality

The collected samples (EM-0 and EM-7) were processed into HnB cigarettes without the addition of any other ingredients. These were subsequently evaluated by five experts from China Tobacco Yunnan Industrial Co. for sensory assessment, they hold professional cigarette sensory evaluation certificates and have been working in this field more than 15 years. In the sensory evaluation process, samples of different groups are randomly assigned to sensory evaluation experts, who score them according to the sensory quality, so as to determine the variability of tobacco raw materials. The evaluation of sensory quality adhered to the criteria established by the Yunyan Tobacco Industry Standard QYNZY.J07.022–2015, titled "New Cigarette Sensory Evaluation Method." In this method, the HnB cigarettes were examined and rated based on six criteria: volume of smoke (10 points), aroma and flavor (30 points), physiological strength (10 points), harmonicity (10 points), irritancy (15 points), and taste (25 points). The final score for each sample was determined by calculating the average of the scores provided by the five experts.

### Untargeted Metabolomics Analysis

During the fermentation process, samples were collected at the onset (0 h), as well as 3 days and 7 days into the fermentation, immediately frozen in liquid nitrogen to halt metabolic activity, and subsequently analyzed for untargeted metabolomics employing LC-MS/MS.

**Metabolite extraction.** A quantity of 25 mg of the sample was weighed into an EP tube, to which 500 μl of extraction solution (methanol: water = 3:1, containing an isotopically-labelled internal standard mixture) was added. Subsequently, the samples were homogenized at 35 Hz for 4 min and sonicated for 5 min in an ice-water bath. This cycle of homogenization and sonication was repeated three times. Following this, the samples were incubated for 1 h at –40°C and centrifuged at 12,000 rpm (RCF = 13,800 ×*g*, R = 8.6 cm) for 15 min at 4°C. The supernatant obtained was then transferred to a new glass vial for subsequent analysis.

**LC‒MS/MS analysis.** LC-MS/MS analyses were conducted using a Vanquish UHPLC system (Thermo Fisher Scientific, USA) equipped with a UPLC HSS T3 column (2.1 mm × 100 mm, 1.8 μm) connected to an Orbitrap Exploris 120 mass spectrometer (Orbitrap MS, Thermo). The mobile phase comprised 5 mmol/l ammonium acetate and 5 mmol/l acetic acid in water (A) and acetonitrile (B). The temperature of the auto-sampler was maintained at 4°C, with an injection volume set at 2 μl.

The Orbitrap Exploris 120 mass spectrometer was utilized for its capacity to perform MS/MS spectra acquisition in information-dependent acquisition (IDA) mode, under the management of acquisition software (Xcalibur, Thermo). In IDA mode, the software persistently monitors the full scan MS spectrum. The ESI source conditions were established as follows: sheath gas flow rate at 50 Arb, auxiliary gas flow rate at 15 Arb, capillary temperature at 320°C, full MS resolution at 60000, MS/MS resolution at 15000, collision energy at 10/30/60 in NCE mode, and spray voltage at 3.8 kV (positive) or –3.4 kV (negative).

### Data Analysis

The raw data obtained via LC‒MS/MS were analyzed via principal component analysis (PCA). Differentially abundant metabolites were screened according to variable importance in the projection (VIP) and *p* value (VIP>1 and *p* < 0.05). OPLS-DA was used to perform confidence tests. All the figures were created using R software (3.3.5) and SIMCA (16.0.2).

## Results

### Results of the Sensory Evaluation

To assess the impact of fermentation on tobacco quality, EM-0 (unfermented tobacco) and EM-7 (tobacco after 7 days of fermentation) samples were selected for the production of HnB cigarettes, which were then subjected to sensory evaluation. The results, presented in Table 1, revealed that the unfermented tobacco (EM-0) exhibited a dry, thin aroma, produced more delicate smoke, and had a moderate smoke volume, achieving an average score of 82 points. In contrast, the tobacco fermented for 7 days (EM-7) displayed enhanced aromatic richness, increased strength, improved moistness, moderate irritation, and a taste that more closely mimicked that of a burning cigarette, resulting in an average score of 84.5. The sensory attributes of the HnB cigarette samples from EM-7 surpassed those of the EM-0 samples, demonstrating that EM fermentation could effectively enhance the sensory quality of HnB cigarettes.

### Qualitative and PCA Results of Metabolites in the Tobacco Samples

**Fig. 1:**
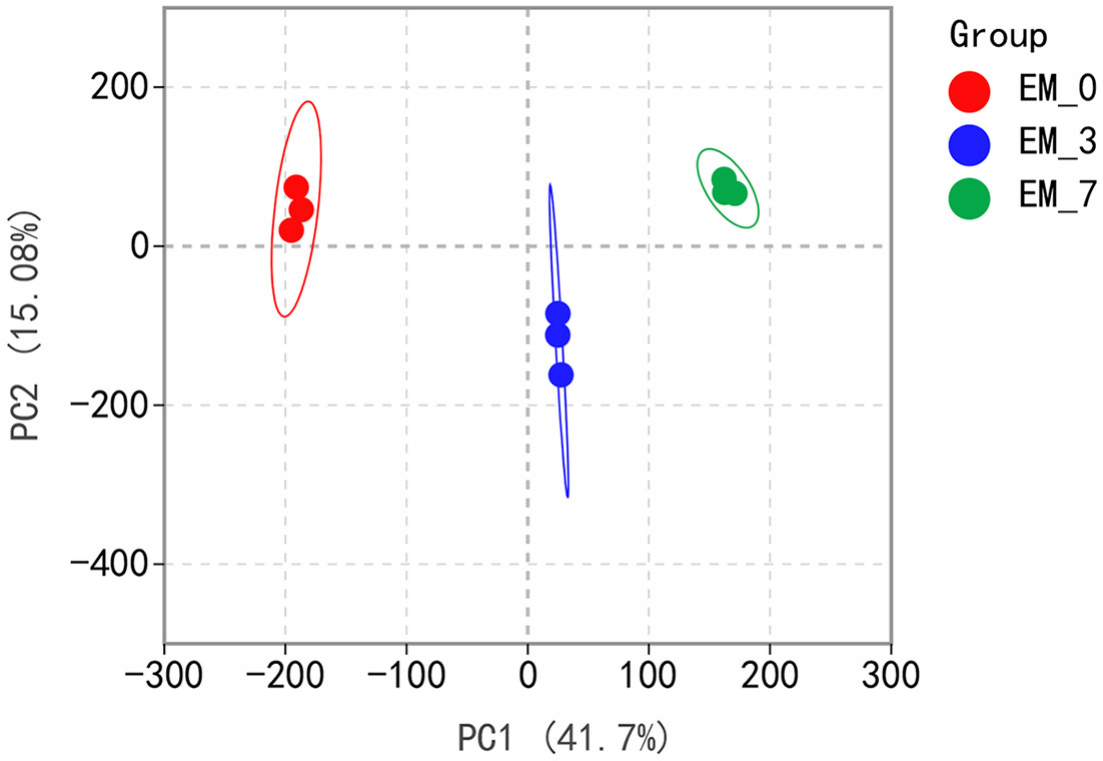
PCA score plots of samples with different fermentation times. The three different colored ovals of red, green and blue indicate different times of fermentation, and the three circles within the ovals indicate three replicates at that fermentation time.

A total of 1191 metabolites in tobacco were identified by LC‒MS/MS analysis (Table S1). PCA revealed the differences in metabolites among the groups (Fig. 1). As we can see, EM-0, EM-3, and EM-7 are clustered into one group, which indicates excellent intragroup reproducibility. However, the intergroup distances were greater, suggesting significant variability between the three groups. The OPLS-DA permutation test diagram (Fig. S1) indicates that the established OPLS-DA model is stable and reliable, there is no over-fitting phenomenon, and the data results are available.

### Results of Differentially Abundant Metabolite Analysis

A total of 416 differentially abundant metabolites(Table S2) were screened from the 1191 metabolites identified, with screening conditions of VIP > 1 and *p* < 0.05 (Fig. S2), where *p*-value was calculated using ANOVA with GraphdPrism software. Obviously, the content of metabolites continued to change during fermentation, among them, the content of 253 metabolites increased gradually with fermentation and 163 metabolites decreased.

**Fig. 2:**
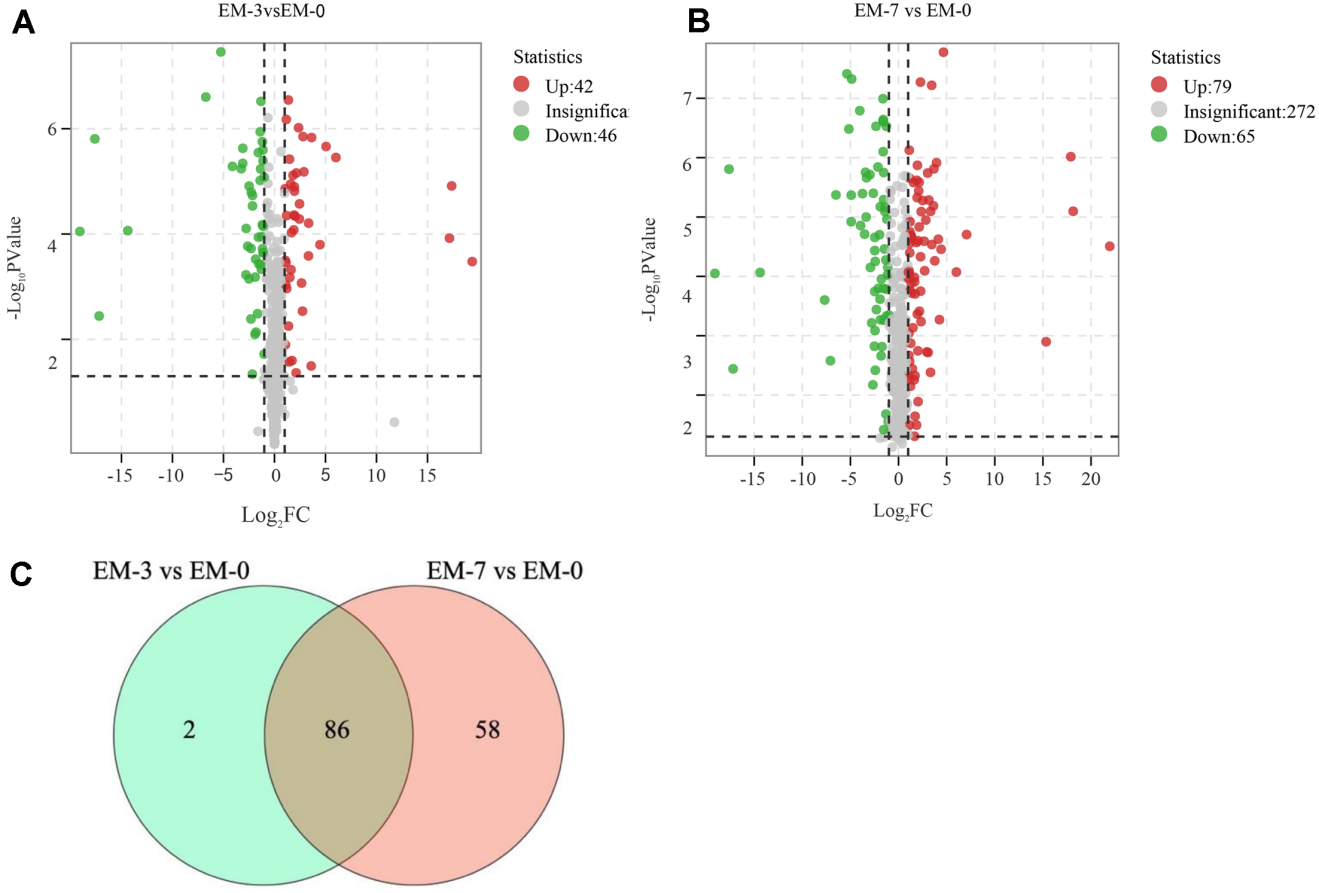
Effects of fermentation duration on the metabolite composition of tobacco leaves. (**A**) Volcano plot of EM-3 vs EM-0. In the figure, red indicates that FC > 2, green indicates that FC < 0.5, and gray indicates that none of the above conditions are met. The sames below. (**B**) Volcano plot of EM-7 vs EM-0; (**C**) Venn diagram of EM-7 vs EM-0 and EM-3 vs EM- 0. There were 144 differential metabolites at 7 days of fermentation compared to fermentation 0 day; 88 differential metabolites at 3 days of fermentation compared to fermentation 0 day. 86 of these metabolites were found in both comparison groups.

To visualize the effect of fermentation duration on tobacco metabolites, we further compared the changes in the content of the above 416 defferential metabolites after 3 and 7 days of fermentation, and the results are shown in Fig. 2 (*p*<0.05 and fold change >2 or < 0.5). Fig. 2A illustrates that a total of 88 metabolites underwent significant changes (42 increased and 46 decreased) in their content after 3 days of fermentation, while after 7 days, the number of significantly changed metabolites rose to 144 (including 79 increased and 65 decreased) as shown in Fig. 2B. This implies that the content of metabolites continues to change as the fermentation proceeds under the action of EM, which is consistent with the trend of substance content changes obtained from Fig. S2. A further comparison of the different metabolites between the two comparative groups revealed that the contents of 86 metabolites changed in both comparison groups throughout the fermentation process, as indicated in Fig. 2C. This suggests that the duration of fermentation has an impact on the composition and content of metabolites in tobacco. These 86 metabolites, detailed in Table S3, mainly include acids, alcohols, esters, and amino acids, among others, and play a role in enhancing tobacco quality. This highlights that tobacco quality is influenced by the duration of fermentation. However, it remains to be determined whether this influence is beneficial or detrimental.

**Fig. 3:**
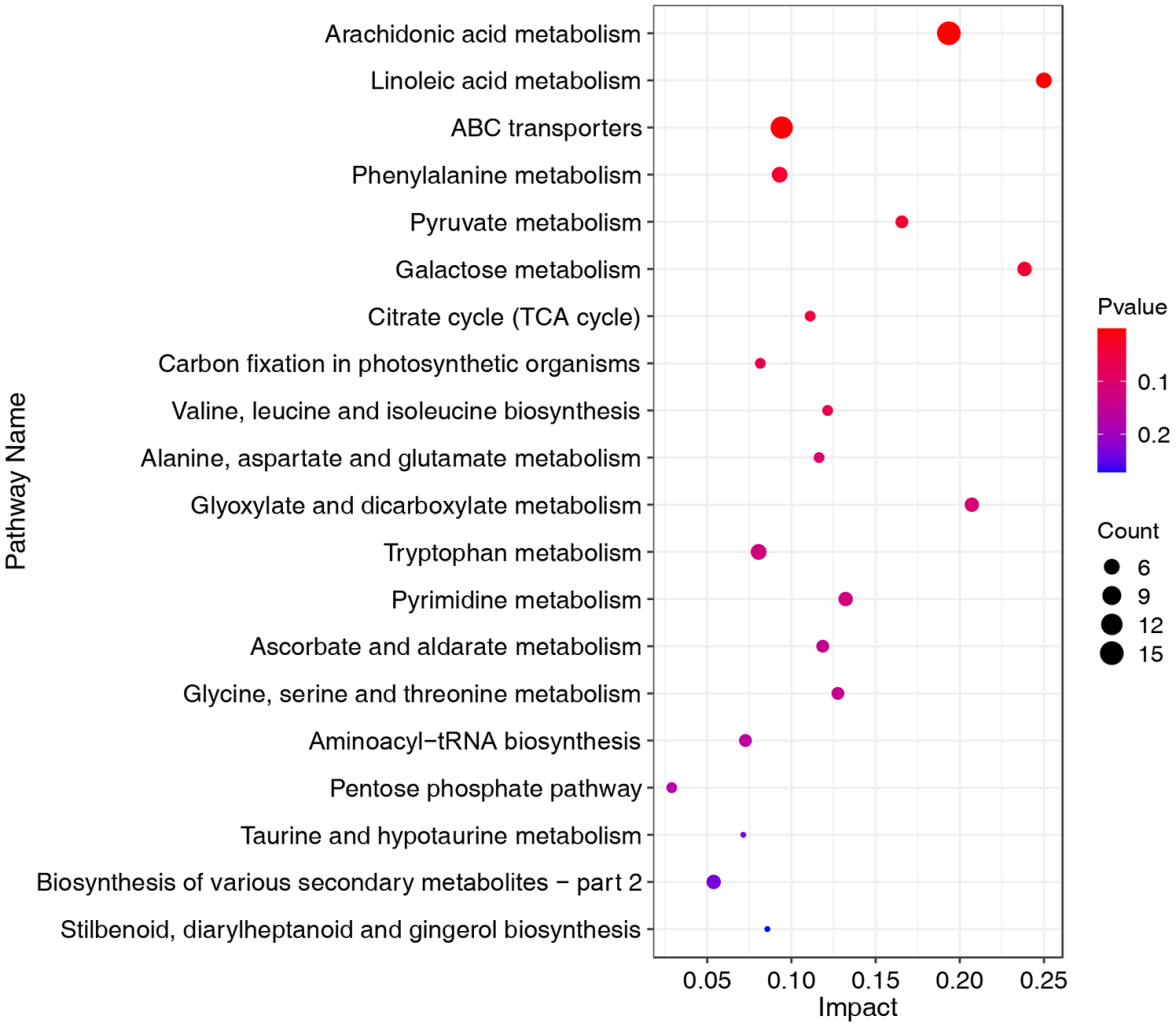
KEGG pathway enrichment results. The y-axis indicates the name of the KEGG metabolic pathway and the x-axis indicates the impact of the pathway, and the significance of the dots indicates the number of DEMs contained in the pathway, and the significance of the dots indicates the number of DEMs contained in the pathway. KEGG pathways are divided into twenty categories and the color of the bars indicates the different metabolic pathway categories.

To further elucidate the mechanism by which microbiota fermentation influences the content of tobacco metabolites and the quality of HnB cigarettes, KEGG pathway enrichment analysis was conducted on the identified differential metabolites. A total of 73 KEGG pathways were identified, with the top 20 pathways demonstrating the most significant enrichment presented in Fig. 3. Beyond basic biological pathways such as the TCA cycle, ABC transfer, and carbon metabolism, the differential metabolites predominantly participate in pathways related to fatty acid metabolism and amino acid metabolism. This includes pathways such as arachidonic acid metabolism, linoleic acid metabolism, phenylalanine metabolism, among others. Additionally, pathways like flavonoid biosynthesis were also enriched (Table S4). These pathways are integral to the biosynthesis of tobacco aroma substances, indicating that microbiota fermentation impacts the quality of tobacco primarily through biological pathways associated with the metabolism of amino acids and fatty acids.

### Analysis of the Aromatic Substances in Tobacco

**Fig. 4:**
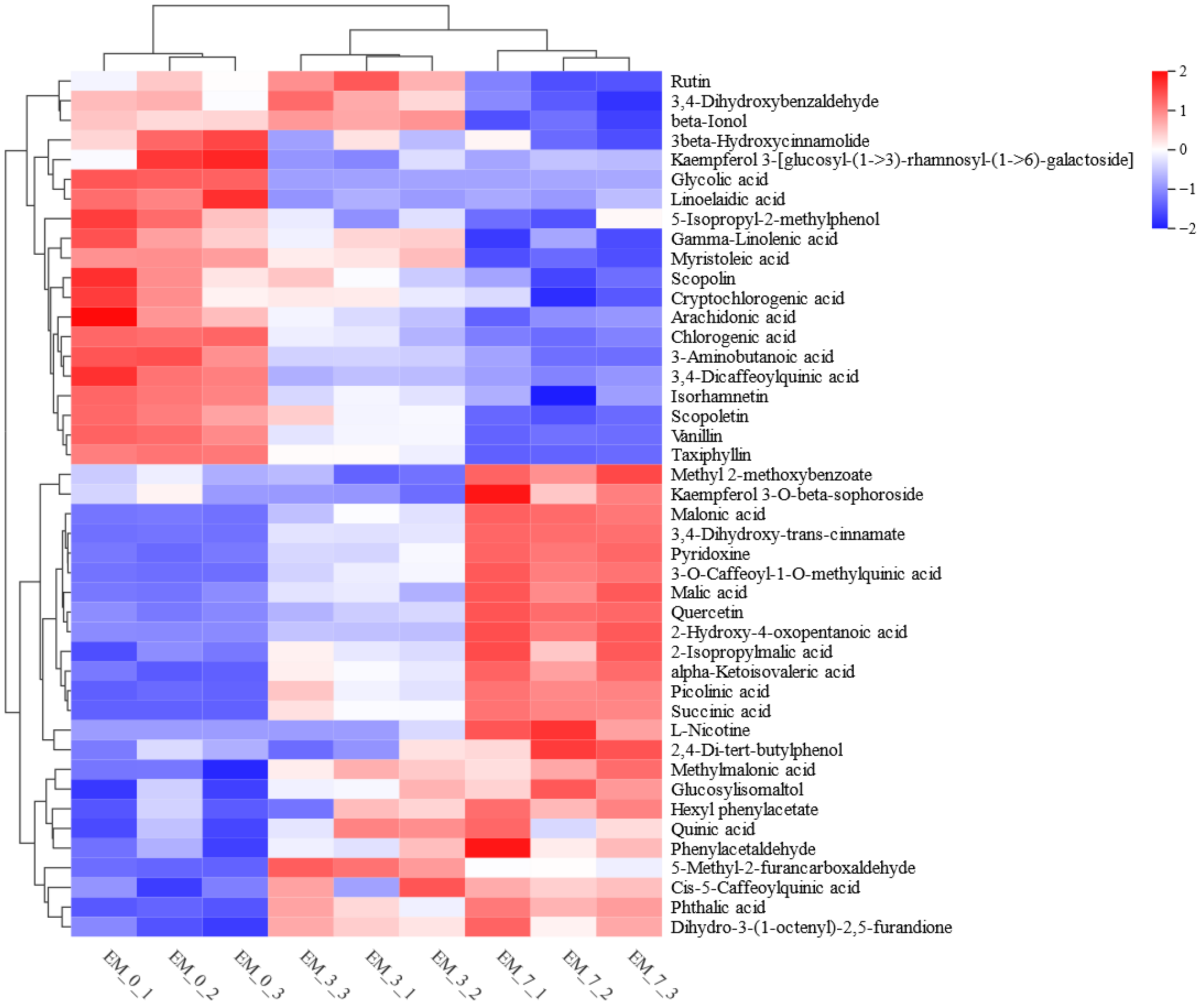
Heatmap of aroma substance content changes during the fermentation process. Expressions are normalized. Blue represents low expression and red represents high expression.

The sensory evaluation results indicated that microbiota fermentation over a period of 7 days led to improvements in the sensory attributes of HnB cigarettes, notably with an increase in smoke volume, aroma richness, strength, and moistness, alongside a reduction in irritation (Table 1). Building on these findings, an analysis was conducted on the composition and content changes of 44 differential metabolites associated with common tobacco aroma substances relevant to tobacco quality. The outcomes are depicted in Fig. 4, illustrating that the content of aroma substances evolved throughout the fermentation process. Notably, the levels of polyphenols such as rutin, scopoletin, chlorogenic acid, cryptochlorogenic acid, and caffeic acid, along with unsaturated fatty acids, including linolenic acid, γ-linolenic acid, and arachidonic acid, exhibited a continuous decrease. Conversely, the content of polyacids like malonic acid, cinnamic acid, malic acid, quinic acid, and succinic acid showed an increase, as did various compounds such as hexyl phenylacetate, furanodione, and pyridoxine. Overall, there was a general decrease in the content of polyphenols and unsaturated fatty acids within the tobacco during fermentation. This reduction in unsaturated fatty acids contributed to the decreased irritation of tobacco, . which was consistent with the results of sensory evaluation that the irritation of the fermented tobacco was reduced (Table 1 ). Meanwhile, the increase in polybasic acids, their derivatives, alcohols, and ketones were conducive to enhancing the richness of the tobacco aroma. The increased content of polybasic acids and their derivatives enhances the aromatic richness of the tobacco when it is smoked, resulting in a milder smoking experience. The increase in the content of alcohols and ketones enhances the quality of the aroma of the tobacco, making it more delicate and elegant, and it also plays a role in enhancing the roundness and cleanliness of the aftertaste of the tobacco when it is smoked. These changes in aroma substance content were largely in alignment with the sensory evaluation results (Table 1), underscoring the significant impact of microbiota fermentation on tobacco quality.

## Discussion

Microorganisms play a crucial role in the aging process of tobacco leaves, offering the benefits of being independent of climate conditions, environmentally friendly, and simple to manage. They reduce the aging cycle and enhance the taste and flavors of tobacco products [26]. Research has identified that the surface microbiota of tobacco leaves predominantly comprises bacterial genera such as *Bacillus*, *Pseudomonas*, *Pantoea*, *Burkholderia*, and *Enterobacter* [27], along with major fungal genera, including *Aspergillus*, *Phom*, *Alternaria*, *Monographella*, and *Cladosporium* [28]. However, bacteria, particularly *Bacillus*, may have a more significant role in enhancing tobacco quality, improving flue gas through the secretion of amylase, acid protease, pectinase, and other hydrolytic enzymes, and producing a broad spectrum of volatile flavor compounds [29]. Employing dominant strains from the tobacco leaf surface or co-fermenting tobacco with multiple genera has been shown to substantially improve tobacco aroma and quality [30], becoming a key trend in tobacco fermentation applications.

The sensory quality of tobacco products, perceived through organoleptic attributes such as aroma and mouthfeel, directly reflects the quality of the cigarette [31]. Sensory evaluation, widely used in the tobacco industry to assess product characteristics, is the principal method for evaluating cigarette production and plays a significant role in determining product pricing. In this study, HnB cigarettes made from fermented tobacco demonstrated superior qualities in terms of aroma richness, strength, moistness, and reduced irritation compared to unfermented samples (Table 1). Similar findings were observed in cigar cigarettes [32], indicating the effectiveness of microbiota fermentation in enhancing tobacco quality and, consequently, the smoking quality of heated cigarettes. The sensory attributes of combustible tobacco are primarily influenced by the metabolites of the tobacco leaf [7]. Thus, further analysis of the composition and content of tobacco metabolites based on sensory results is essential for a deeper understanding of the variations in tobacco quality.

Untargeted metabolomics was utilized to analyze changes in the content of various tobacco aroma substances, including polyphenols, fatty acids, polybasic acids, and their derivatives, alcohols, ketones, and esters, as shown in Fig. S1. Notably, the levels of rutin, chlorogenic acid, and caffeic acid significantly decreased, aligning with findings from previous studies [33]. This reduction might be attributed to the transformation of polyphenols from glycosides or esters during fermentation, resulting in a lower content post-fermentation. Furthermore, a significant decrease in the content of unsaturated fatty acids, such as linoleic acid and γ-linolenic acid, was observed, mirroring results reported elsewhere [34]. It was suggested that a reduced level of unsaturated fatty acids could be instrumental in lowering irritation and enhancing the smoking quality of tobacco. This hypothesis is supported by the sensory evaluation outcomes of this study, which indicated a reduction in tobacco irritation following fermentation (Table 1), thereby underscoring the efficacy of microbiota fermentation in mitigating irritation and improving tobacco quality. Moreover, an increase in the content of polybasic acids and their derivatives, including malic, succinic, malonic, and cinnamic acids, was noted, corroborating the findings of Zhou *et al*. [35]. It has been demonstrated that higher concentrations of polybasic acids in tobacco contribute to a more flavorful experience, reduced irritation, and increased mouth comfort during smoking[8].

Similarly, higher levels of ketones, aldehydes, esters, and furans are associated with a richer tobacco flavor, enhancing sweet, floral, and caramelized aromas, as well as a full-bodied, mellow, and delicate taste in flue-cured tobacco [36, 37]. However, this study also identified substances that decreased in content following fermentation (Fig. 4), which is inferred to be the result of microbiota effects. Compared to the use of single or dual microorganisms in prior studies, employing microbial consortia for fermenting tobacco leaves may offer enhanced benefits. Firstly, the microbial consortia, enriched from the surfaces of tobacco leaves, include a diverse array of microorganisms, including bacteria and fungi. This diversity facilitates complex metabolic interactions [38], producing a wider variety of flavor compounds and enriching the flavor profile of fermented tobacco [39, 40]. Secondly, the microbial consortia are naturally occurring in the tobacco environment and have adapted to it, potentially aligning more closely with the natural ecosystem's balance during fermentation [41]. This adaptation aids in maintaining the stability of the fermentation system and enables more effective metabolic activity, utilizing the nutrients from the tobacco leaf and influencing the final quality of the tobacco [42]. Despite the reduction in polyphenol content in fermented tobacco, the increased levels of various aroma substances, primarily polybasic acids, effectively enhanced the overall smoking quality of tobacco, as confirmed by sensory evaluation results. Thus, microbiota fermentation has been demonstrated to significantly improve the sensory quality of HnB cigarettes.

KEGG pathway enrichment analysis revealed the underlying mechanisms associated with changes in tobacco during fermentation. In this investigation, 415 differential metabolites were classified into 73 KEGG pathways (Table S4), indicating that the microbiota affecting tobacco quality triggers complex biological reactions. Predominantly, the metabolism of fatty acids and amino acids emerged as the main pathways (Fig. 3), aligning with the findings of Mellema *et al*. [43] that fatty acid metabolism and amino acid metabolism significantly contribute to the enhancement of tobacco quality. This evidence further underscores the close relationship between microbiota fermentation in enhancing the quality of HnB cigarettes and the metabolic pathways of fatty acids and amino acids. Additionally, pathways involving starch and sucrose metabolism have been identified to reduce irritation in roasted tobacco [44, 45], and the flavonoid biosynthesis pathway plays a crucial role in generating aroma substances. These quality-related pathways were enriched in the current study (Table S4), with the differential metabolites positioned upstream in these pathways. Based on the alterations in their content, it is hypothesized that these pathways exert enhancing effects, thereby contributing to quality improvement.

Artificial fermentation presents a promising approach to enhancing tobacco quality, characterized by its reduced time frame, cost-efficiency, and high effectiveness. Building on the findings of prior research, this study utilized microbiota enriched from the surface of tobacco leaves to ferment raw flue-cured tobacco designated for HnB cigarettes. The outcomes indicated a notable improvement in the smoking quality of HnB cigarettes attributed to the microbiota, underscoring the fermentation method's significant potential for industrial application. Nonetheless, the untargeted metabolomics technology employed in this investigation faced limitations in detecting the full range of tobacco aroma substances. This restriction poses a challenge for a thorough evaluation of the sensory quality of HnB cigarettes, highlighting a need for advancements in the associated research methodologies.

## Conclusion

Our research utilized microbiota enriched from the surface of tobacco leaves to ferment raw flue-cured tobacco intended for HnB cigarettes, with sensory evaluations demonstrating a significant enhancement in cigarette quality. Untargeted metabolomics analysis revealed a decrease in polyphenols and unsaturated fatty acids, alongside an increase in the content of polyacids, alcohols, ketones, furans, and other aroma substances within the fermented tobacco. These changes were closely associated with the improvement of the sensory quality of the cigarettes. Further analysis elucidated that microbial fermentation for HnB cigarettes is linked to various biological pathways, among which the metabolism of fatty acids and amino acids plays pivotal roles. These findings introduce a novel approach to tobacco fermentation and offer a theoretical basis for exploring the mechanisms behind tobacco quality enhancement.

## Supplemental Materials

Supplementary data for this paper are available on-line only at http://jmb.or.kr.



## Figures and Tables

**Table 1:** Sensory evaluation of HnB cigarettes before and after fermentation.

Samples	Volume of smoke (10–0)	Aroma and flavor (30–0)	Physiological strength (10–0)	Harmony (10–0)	Irritancy (15–0)	Taste (25–0)	Total (100–0)
EM-0	8.00 ± 0.2	22.86 ± 0.22	8.02 ± 0.23	7.98 ± 0.15	13.10 ± 0.14	22.10 ± 0.14	82.06 ± 0.30
EM-7	8.56 ± 0.11*	24.14 ± 0.26**	9.12 ± 0.28**	8.62 ± 0.28*	12.46 ± 0.36	22.30 ± 0.21	85.2 ± 0.31**

The data in the table are the average of the scores given by the five experts. *Means *p* < 0.05, **means *p* < 0.01

## References

[1] Kim YH, An YJ (2020). Development of a standardized new cigarette smoke generating (SNCSG) system for the assessment of chemicals in the smoke of new cigarette types (heat-not-burn (HNB) tobacco and electronic cigarettes (E-Cigs)). Environ. Res..

[2] Simonavicius E, McNeill A, Shahab L, Brose LS (2019). Heat-not-burn tobacco products: a systematic literature review. Tob. Control.

[3] Li X, Luo Y, Jiang X, Zhang H, Zhu F, Hu S (2019). Chemical analysis and simulated pyrolysis of tobacco heating system 2.2 compared to conventional cigarettes. Nicotine Tob. Res..

[4] Farsalinos KE, Yannovits N, Sarri T, Voudris V, Poulas K (2018). Nicotine delivery to the aerosol of a heat-not-burn tobacco product: comparison with a tobacco cigarette and E-cigarettes. Nicotine Tob. Res..

[5] Znyk M, Jurewicz J, Kaleta D (2021). Exposure to heated tobacco products and adverse health effects, a systematic review. Int. J. Environ. Res. Public Health.

[6] Ning Y, Mai J, Hu BB, Lin ZL, Chen Y, Jiang YL (2023). Study on the effect of enzymatic treatment of tobacco on HnB cigarettes and microbial succession during fermentation. Appl. Microbiol. Biotechnol..

[7] Popova V, Ivanova T, Prokopov T, Nikolova M, Stoyanova A, Zheljazkov VD (2019). Carotenoid-related volatile compounds of tobacco (*Nicotiana tabacum* L.) essential oils. Molecules.

[8] Yin F, Karangwa E, Song S, Duhoranimana E, Lin S, Cui H (2019). Contribution of tobacco composition compounds to characteristic aroma of Chinese faint-scent cigarettes through chromatography analysis and partial least squares regression. J. Chromatogr. B Analyt. Technol. Biomed. Life Sci..

[9] Weeks WW, Sisson VA, Chaplin JF (1992). Differences in aroma, chemistry, solubilities, and smoking quality of cured flue-cured tobaccos with aglandular and glandular trichomes. J. Agric. Food Chem..

[10] Zheng T, Zhang Q, Li P, Wu X, Liu Y, Yang Z (2022). Analysis of microbial community, volatile flavor compounds, and flavor of cigar tobacco leaves from different regions. Front. Microbiol..

[11] Liu F, Zhao Z, Zhao M (2016). Detection and quantitative analysis of dominant bacteria on aging flue-cured tobacco leaves. Agric. Sci. Technol..

[12] Wen C, Zhang Q, Zhu P, Hu W, Jia Y, Yang S (2023). High throughput screening of key functional strains based on improving tobacco quality and mixed fermentation. Front. Bioeng. Biotechnol..

[13] Hu B, Gu K, Gong J, Zhang K, Chen D, He X (2021). The effect of flue-curing procedure on the dynamic change of microbial diversity of tobaccos. Sci. Rep..

[14] Zhang Q, Kong G, Zhao G, Liu J, Jin H, Li Z (2023). Microbial and enzymatic changes in cigar tobacco leaves during air-curing and fermentation. Appl. Microbiol. Biotechnol..

[15] Liu F, Wu Z, Zhang X, Xi G, Zhao Z, Lai M (2021). Microbial community and metabolic function analysis of cigar tobacco leaves during fermentation. Microbiologyopen.

[16] Banožić M, Jokić S, Ačkar Đ, Blažić M, Šubarić D (2020). Carbohydrates-key players in tobacco aroma formation and quality determination. Molecules.

[17] Liu T, Guo S, Wu C, Zhang R, Zhong Q, Shi H (2022). Phyllosphere microbial community of cigar tobacco and its corresponding metabolites. Front. Microbiol..

[18] Zheng T, Zhang Q, Wu Q, Li D, Wu X, Li P (2022). Effects of inoculation with acinetobacter on fermentation of cigar tobacco leaves. Front. Microbiol..

[19] Xu Q, Li S, Huang S, Mao D (2021). Review on tobacco-derived microorganisms and its application. J. Light Ind..

[20] Zheng Z (2021). Study on the microbial diversity of tobacco and its effect on the fermentation quality of tobacco. Master.

[21] Ma L, Wang Y, Wang X, Lü X (2023). Solid-state fermentation improves tobacco leaves quality via the screened *Bacillus subtilis* of simultaneously degrading starch and protein ability. Appl. Biochem. Biotechnol..

[22] Dai J, Dong A, Xiong G, Liu Y, Hossain MS, Liu S (2020). Production of highly active extracellular amylase and cellulase from *Bacillus subtilis* ZIM3 and a recombinant strain with a potential application in tobacco fermentation. Front. Microbiol..

[23] Li J, Zhao Y, Qin Y, Shi H (2020). Influence of microbiota and metabolites on the quality of tobacco during fermentation. BMC Microbiol..

[24] Zhou J, Yu L, Zhang J, Zhang X, Xue Y, Liu J (2020). Characterization of the core microbiome in tobacco leaves during aging. Microbiologyopen.

[25] Ren M, Qin Y, Zhang L, Zhao Y, Zhang R, Shi H (2023). Effects of fermentation chamber temperature on microbes and quality of cigar wrapper tobacco leaves. Appl. Microbiol. Biotechnol..

[26] Edgar RC (2013). UPARSE: highly accurate OTU sequences from microbial amplicon reads. Nat. Methods.

[27] Huang J, Yang J, Duan Y, Gu W, Gong X, Zhe W (2010). Bacterial diversities on unaged and aging flue-cured tobacco leaves estimated by 16S rRNA sequence analysis. Appl. Microbiol. Biotechnol..

[28] Liu F, Zhang X, Wang M, Guo L, Yang Y, Zhao M (2020). Biosorption of sterols from tobacco waste extract using living and dead of newly isolated fungus *Aspergillus* fumigatus strain LSD-1. Biosci. Biotechnol. Biochem..

[29] Gong Y, Li J, Deng X, Chen Y, Chen S, Huang H (2023). Application of starch degrading bacteria from tobacco leaves in improving the flavor of flue-cured tobacco. Front. Microbiol..

[30] Ye J, Zhang Z, Yan J, Hao H, Liu X, Yang Z (2017). Degradation of phytosterols in tobacco waste extract by a novel *Paenibacillus* sp. Biotechnol. Appl. Biochem..

[31] Carpenter CM, Wayne GF, Connolly GN (2007). The role of sensory perception in the development and targeting of tobacco products. Addiction.

[32] Zong P, Hu W, Huang Y, An H, Zhang Q, Chai Z (2023). Effects of adding cocoa fermentation medium on cigar leaves in agricultural fermentation stage. Front. Bioeng. Biotechnol..

[33] Liu H, Hu L, Yan K, Long M, Shan X, Zi W (2015). Study on changes in. internal chemical components of flue-cured tobacco in process of natural alcoholization. Acta Agric. Jiangxi..

[34] Hu W, Cai W, Zheng Z, Liu Y, Luo C, Xue F (2022). Study on the. chemical compositions and microbial communities of cigar tobacco leaves fermented with exogenous additive. Sci. Rep..

[35] Zhou G, Chen J, Kong L, Yang S, Xia Q, Liu J (2014). Analysis of aroma components of diverse tobacco materials in main tobacco areas of Yunnan. Southwest China J. Agric. Sci ..

[36] Ding Y, Zhu L, Liu S, Yu H, Dai Y (2013). Analytical method of free and conjugated neutral aroma components in tobacco by solvent extraction coupled with comprehensive two-dimensional gas chromatography-time-of-flight mass spectrometry. J. Chromatogr. A..

[37] Zhang Q, Ma Z, Meng Q, Li D, Ding Z (2021). Key aroma compounds and metabolic profiling of *Debaryomyces hansenii* L1-1-fermented flos sophorae. J. Food Biochem..

[38] Landis EA, Fogarty E, Edwards JC, Popa O, Eren AM, Wolfe BE (2022). Microbial diversity and interaction specificity in Kombucha tea fermentations. mSystems.

[39] Tang Q, Liu T, Teng K, Xiao Z, Cai H, Wang Y (2023). Microbial interactions and metabolisms in response to bacterial wilt and black shank pathogens in the tobacco rhizosphere. Front. Plant Sci..

[40] Zhou M, Sun C, Dai B, He Y, Zhong J (2023). Intercropping system modulated soil-microbe interactions that enhanced the growth and quality of flue-cured tobacco by improving rhizospheric soil nutrients, microbial structure, and enzymatic activities. Front. Plant Sci..

[41] Gao J, Uwiringiyimana E, Zhang D (2023). Microbial composition and diversity of the tobacco leaf phyllosphere during plant development. Front. Microbiol..

[42] Zhang G, Zhao L, Li W, Yao H, Lu C, Zhao G (2023). Changes in physicochemical properties and microbial community succession during leaf stacking fermentation. AMB Express.

[43] Mellema S, Eichenberger W, Rawyler A, Suter M, Tadege M, Kuhlemeier C (2002). The ethanolic fermentation pathway supports respiration and lipid biosynthesis in tobacco pollen. Plant J..

[44] Pan D, Sun M, Wang Y, Lv P, Wu X, Li QX (2018). Characterization of nicotine catabolism through a novel pyrrolidine pathway in *Pseudomonas* sp. S-1. J. Agric. Food Chem..

[45] Li J, Yi F, Chen G, Pan F, Yang Y, Shu M (2021). Function enhancement of a metabolic module via endogenous promoter replacement for *Pseudomonas* sp. JY-Q to degrade nicotine in tobacco waste treatment. Appl. Biochem. Biotechnol..

